# Supplementary material for: Study of shear-plastic slip mechanism based on TC4 titanium alloy
Source: PLoS One. 2026 Jan 23;21(1):e0338815. doi: 10.1371/journal.pone.0338815 (PMC12829965; doi:10.1371/journal.pone.0338815)
Supplement: Attachment — Submitted filename: PONE-D-25-26088-Response-to-Reviewers.doc [file pone.0338815.s002.doc]

**Original Manuscript ID:** PONE-D-25-26088

**Original Manuscript Title:** “Study Of Shear-Plastic Slip Mechanism Based On TC4 Titanium Alloy”

**To:** Plos One

, Editor, Gaurav Ashok Bhaduri, PhD

**Re:** Response to reviewers

Dear Editor,

Thank you for allowing a revision of our manuscript, with an opportunity to address the reviewers’ comments. **The submitted manuscript is the revised and resubmitted version of** P**ONE-D-25-26088.**

We are uploading **(a)** a cover letter for the revised manuscript, which describes how we have incorporated the reviewers' comments, **(b)** our point-by-point response to all the comments from the reviewers (response-to-reviewers), and **(c)** an updated manuscript with red highlighting indicating changes.

Dear Reviewers,

Thanks very much for your valuable comments on this manuscript. We believe that your comments strongly improved the quality of our manuscript and increased its research contribution. We have made modifications and noted changes with highlights for these comments in the corresponding locations of our revision manuscript. Thank you again for your comments. We respond to these comments in the next few pages.

Best regards,

The authors < Bo Hu, Zichuan Zou, Pengfei Tian, Nian Xiao, Sen Yuan, Xianfeng Zhao >.

**Reviewer#1 Comments**

**Concern #1:** The paper identifies the tool rake angle (≤15°) and tool tip radius as key factors influencing the stagnation point. It is recommended to supplement a quantitative analysis of stress triaxiality and Lode angle under different cutting speeds, comparing the applicability differences of J-C, H-M, and MMC fracture criteria under dynamic loading conditions. Experimental validation can reference research on the correlation between wear mechanisms and stress distribution of high-entropy alloy-coated tools in micro-cutting TC4 titanium alloy.

**Author response:** The stress distribution under varying cutting speeds has been comprehensively addressed in our separate publication, entitled "Mechanical analysis before the steady-state cutting of TC4 titanium alloy". Furthermore, as our research has primarily utilized the Johnson-Cook fracture criterion, we lack the foundational data and expertise to rigorously evaluate alternative models such as the Hosford-Coulomb (H-M) or the Modified Mohr-Coulomb (MMC) criteria at this time. We acknowledge this limitation in the current study.

**Concern #2:** The current model visualizes the slip-line field through secondary development in SOLIDWORKS. It is suggested to introduce a multi-scale finite element simulation approach: at the macroscopic scale, coupling the thermomechanical constitutive model for diffusion bonding of TC4 titanium alloy while considering the effect of initial welding thermal-mechanical coupling on subsequent cutting deformation; at the microscopic scale, embedding the dislocation density evolution equation to characterize the synergistic effects of α→β phase transformation and void toughening mechanisms within the adiabatic shear band. A comparative analysis of prediction error ranges between the traditional slip-line field theory and the improved model is needed.

**Author response:** We are grateful for the insightful comment raised by the reviewer. It is important to note that we have already recognized the value of this approach and have initiated a separate research project utilizing a novel molecular dynamics framework to tackle this specific challenge. Although this falls outside the immediate scope of the present manuscript, the initial results are highly encouraging, and we anticipate that it will yield a comprehensive understanding in the foreseeable future.

Concern #3: Regarding the applicability threshold of the 15° rake angle in the dead-zone model, a phased validation strategy is recommended: for the low strain rate phase (<0.1 s⁻¹), reference in-situ observations of grain refinement and dynamic recrystallization behavior during TC4 hot rolling; for the high strain rate phase (>1 s⁻¹), incorporate the microvoid evolution patterns in adiabatic shear bands under one-dimensional strain compression to calibrate the correlation between slip-band inclination and temperature field distribution.

**Author response:** We are grateful for the insightful comment raised by the reviewer. It is important to note that we have already recognized the value of this approach and have initiated a separate research project utilizing a novel molecular dynamics framework to tackle this specific challenge. Although this falls outside the immediate scope of the present manuscript, the initial results are highly encouraging, and we anticipate that it will yield a comprehensive understanding in the foreseeable future.

**Concern #4:** The paper focuses on fundamental mechanisms, but should supplement an evaluation of TC4 cutting parameters' effects on the surface integrity of aerospace components. For example, comparing grain boundary migration differences between conventional rolling processes (ε = 0.0001~0.1 s⁻¹) and cutting-induced dynamic strain rates, quantifying the impact of dead-zone size on residual stress and fatigue life of the machined surface.

**Author response:** We thank the reviewer for their pertinent suggestion. This very issue has been incorporated as a key objective in our recent research project. In our subsequent work, we plan to employ a micro-mechanical approach to the cutting process to systematically investigate the influence of cutting parameters on the formation mechanisms of subsurface defects. The findings will be rigorously validated using advanced characterization techniques, including Scanning Electron Microscopy (SEM) and Electron Backscatter Diffraction (EBSD).

**Reviewer#2 Comments**

In general, this manuscript is suggested to be accepted for publication after minor revision.It is recommended to take notice the following:

**Author response:** We extend our sincere gratitude for your encouraging comments on our work. We are committed to pursuing further rigorous and in-depth research in this field.

**Concern #1:** A little bit of rearranging of the abstract is needed maybe. Abstract is usually divided into four parts WHY, WHAT, HOW, and main conclusions.
WHY → This section usually contains one or two lines mainly defining what is the objective of the study or this work was done.
WHAT → This is the main portion of the abstract. It contains what was done. Like what simulations have been performed what kind of parametric studies are done to support the WHY section.
HOW → In this section you will define how u have achieved the WHAT section points. What kind of methodologies you have utilized to achieve the goals defined in WHAT section?
Usually at the end you will include one or two lines that how it is going to benefit the scientific community or what are the readership of this paper.

**Author response:** We would like to express our sincere gratitude for your review of our manuscript. In response to your comments on the abstract, we have revised it accordingly. The changes have been highlighted in red within the revised manuscript. Additionally, we have expanded the Conclusion section to include a discussion on future research prospects.

The stagnation point and dead metal zone during the cutting process directly influence chip formation and stress distribution, while the stress distribution during machining determines the direction of plastic slip in the material during cutting. This study addresses the current theoretical gap in understanding the dead metal zone and stagnation point during cutting. The cutting process of TC4 titanium alloy is analyzed by dividing it into two distinct stages: the tool-edge contact stage and the tool rake face contact stage. Based on the stress and pressure distributions in these two stages, a slip-line field model incorporating the dead metal zone is developed. The slip-line field is computed by solving the Cauchy problem and visualized via the SOLIDWORKS secondary development interface. The model for the dead metal zone and stagnation point was validated through finite element simulations under orthogonal cutting conditions. Subsequent machining experiments confirmed the slip-line field model and revealed the plastic slip behavior during TC4 cutting. Results indicate that the dead metal zone model provides higher predictive accuracy when the tool rake angle is ≤15°; the stagnation point is most significantly influenced by the tool rake angle and the cutting edge radius; and the proposed slip-line field model, which includes the dead metal zone, more accurately reflects the actual plastic slip phenomena in cutting. In conclusion, the dead metal zone model, stagnation point model, and slip-line field model collectively elucidate the cutting mechanism in the elasto-plastic stage, establishing a foundation for subsequent research on tool wear, chip formation, and machined surface quality.

**Concern #2**: The objective of this work should be modified in the end of introduction.

**Author response:** We extend our sincere gratitude for your review of our manuscript. As suggested, we have further clarified the research objectives in the final paragraph of the Introduction. The revisions have been highlighted in red in the resubmitted manuscript.

**Concern #3**: What is the criteria for selection of parameters given in Table 1?

**Author response:** We sincerely thank the reviewer for their assessment of our manuscript. Table 1 presents the parameters selected for simulating the orthogonal cutting process.

**Concern #4**: Provide the reference for the equations you used.

**Author response:** We sincerely appreciate the reviewer's valuable feedback. In response, we have thoroughly referenced all equations in the manuscript whose sources were previously unclear. These additions have been highlighted in red in the revised version.

**Concern #5**: Provide the details about the FEM model developed.

**Author response:** We sincerely thank the reviewer for this valuable feedback. In response, we have added a comprehensive elaboration of the simulation procedure following Table 1 in the manuscript. The newly added text has been highlighted in red for the reviewer's convenience.

Modeling is done by orthogonal cutting, the tool is selected from the library with its carbide tool, the size is set according to Table 1, the workpiece is selected from the library with its own TC4, the size is set to width (5mm) * height (10mm), the tool mesh is divided into 700 cells, the workpiece mesh is divided into 25 cells, the minimum number of steps is set to 10, the friction coefficient is set to 0.2, and the damage model used is the J-C model.

**Concern #6:** Add more information about the results from dead metal zone morphology for different cutting parameters.

**Author response:** We are grateful to the reviewer for this insightful observation. It is correct that our preliminary simulations encompassed a broader spectrum of rake angles (-20° to 25°). A critical comparison with our theoretical framework, however, demonstrated conclusive validation specifically for the 0° to 15° range. We made a strategic decision to concentrate the manuscript on this well-validated regime to provide a clear and robust narrative. The full dataset, including dead zone morphologies for all simulated angles, was comprehensively documented in the author's foundational Master's thesis [1], which this current work builds upon.

[1] Hu Bo. Study on Cutting Force of Titanium Alloy TC4 Based on Slip Line Field [D]. Guizhou University, 2022. DOI:10.27047/d.cnki.ggudu.2022.000441.

**Concern #7**:It is hard to follow the Slip line field for different rake angles, explain more.

**Author response:** We thank the reviewer for this comment. We acknowledge that the evolution of the slip-line field is indeed a complex process. A more detailed discussion of the underlying physical mechanisms is provided in the first author's Master's thesis [1] and in reference [29] cited in the manuscript.

[1] Hu Bo. Study on Cutting Force of Titanium Alloy TC4 Based on Slip Line Field [D]. Guizhou University, 2022. DOI:10.27047/d.cnki.ggudu.2022.000441.

**Concern #8:** A short subsection on “Future Research Directions” in the conclusion.

**Author response:**We sincerely thank the reviewer for this valuable suggestion. In response, we have added a section outlining future research prospects to the conclusion of the manuscript. These modifications have been highlighted in red in the revised version for your convenience.

**Reviewer#3 Comments**

**Concern #1:** The title has a weak correlation with keywords, and the keywords should be optimized.

**Author response:** We sincerely thank the reviewer for this valuable feedback. In response, we have carefully revised the keywords in the manuscript to better reflect the core themes and enhance the paper's relevance and discoverability.

**Concern #2:** The abstract only describes the model construction process but does not clarify the research objectives.

**Author response:** We sincerely thank the reviewer for this valuable suggestion. In response, we have revised the abstract to more clearly articulate the research objectives of this study. The modifications have been highlighted in red in the revised manuscript for your convenience.

**Concern #3:** The paper only verifies the accuracy of the dead metal zone model when the rake angle ≤ 15°, but does not clearly explain the specific reasons for model failure when the rake angle > 15°.

**Author response:** We are grateful to the reviewer for this insightful observation. It is correct that our preliminary simulations encompassed a broader spectrum of rake angles (-20° to 25°). A critical comparison with our theoretical framework, however, demonstrated conclusive validation specifically for the 0° to 15° range. We made a strategic decision to concentrate the manuscript on this well-validated regime to provide a clear and robust narrative. The full dataset, including dead zone morphologies for all simulated angles, was comprehensively documented in the author's foundational Master's thesis [1], which this current work builds upon.

[1] Hu Bo. Study on Cutting Force of Titanium Alloy TC4 Based on Slip Line Field [D]. Guizhou University, 2022. DOI:10.27047/d.cnki.ggudu.2022.000441.

**Concern #4:** The experiment only uses a single working condition to verify the slip-line field, without covering different rake angles.

**Author response:** We sincerely thank the reviewer for this insightful comment. We acknowledge that the current analysis is primarily based on a single-factor investigation. In our future work, we plan to incorporate multi-physics simulations that account for interacting mechanisms to enable a comprehensive multi-factor analysis, thereby strengthening the overall study.

**Concern #5:** Some research work can be useful in the introduction. Effect of polymeric aluminum chloride waste residue and citric acid on the properties of magnesium oxychloride cement.

**Author response:** We thank the reviewer for the valuable suggestion. The relevant references have been incorporated into the Introduction as recommended.

**Concern #6**:The first point in the conclusion highly repeats the content in the middle of the abstract, both describing the model construction process.

**Author response:** We sincerely thank the reviewer for this constructive feedback. We have reorganized the content to improve the logical flow and clarity of the manuscript. The revised sections have been highlighted in red for your convenience.

**Concern #7:**The conclusion does not clearly propose process optimization suggestions for cutting TC4 titanium alloy.

**Author response:** We are grateful for the valuable suggestion. In response, we have strengthened the Conclusion section through further deliberation and explicitly put forward the following recommendations for optimizing the cutting process of TC4 titanium alloy:

1. To mitigate work hardening and stress concentration, it is advisable to reduce the tool rake angle and cutting edge radius. This adjustment enhances tool sharpness, thereby improving cutting performance.

2. In future work, we plan to implement a molecular dynamics model to investigate the mechanical behavior of TC4 from a micro-mechanical perspective. This approach will allow us to analyze phenomena such as grain dislocation and sub-surface damage during cutting, ultimately facilitating further optimization of the machining process.

**Reviewer#4 Comments**

**The study addresses slip-line field theory in TC4 titanium alloy cutting, focusing on dead metal zone and stagnation point, but the novelty is limited since many classical models already exist.**

**Author response:** We thank the reviewer for this valuable feedback. We acknowledge that the derivations in this manuscript are grounded in established classical theories, with a primary focus on the dead metal zone and the stagnation point. As such, the work is modest in its fundamental novelty. Nevertheless, the theoretical results derived from classical mechanical analysis have been experimentally validated, which we believe demonstrates the reliability and explanatory power of the presented approach.

**Concern #1:** Theoretical derivations use assumptions on stress distribution and elastic–plastic transition without rigorous justification, making the model appear weak in scientific grounding.

**Author response:** We thank the reviewer for raising this pertinent question. In developing our theoretical derivations from existing models, we needed to introduce certain boundary conditions to complete the framework. We also acknowledge that our current experimental setup has limitations that prevent a full experimental validation of all theoretical aspects at this stage. To address this, we plan to employ molecular dynamics simulations in future work to further investigate the stress distribution and elastoplastic transition behavior. A more detailed step-by-step theoretical derivation is already provided in reference [29] of the manuscript.

**Concern #2:** Simulation work through SolidWorks is constrained to narrow parameter ranges and lacks quantitative validation; advanced FEM platforms would provide more reliable results.

**Author response:** We thank the reviewer for this expert suggestion. We fully agree that utilizing advanced simulation platforms can lead to more reliable results. In our subsequent research, we plan to incorporate multiple simulation approaches for cross-validation, with a specific focus on employing molecular dynamics simulations to gain deeper insights into the underlying mechanisms from a micro-scale perspective. In addition, it is correct that our preliminary simulations encompassed a broader spectrum of rake angles (-20° to 25°). A critical comparison with our theoretical framework, however, demonstrated conclusive validation specifically for the 0° to 15° range. We made a strategic decision to concentrate the manuscript on this well-validated regime to provide a clear and robust narrative. The full dataset, including dead zone morphologies for all simulated angles, was comprehensively documented in the author's foundational Master's thesis [1], which this current work builds upon.

[1] Hu Bo. Study on Cutting Force of Titanium Alloy TC4 Based on Slip Line Field [D]. Guizhou University, 2022. DOI:10.27047/d.cnki.ggudu.2022.000441.

**Concern #3:** Differences between theoretical predictions and simulation values of the dead metal zone are significant, and the explanation for these discrepancies is not convincing.

**Author response:** We thank the reviewer for this guiding comment. We acknowledge that the current simulation software has limitations in providing intuitive visualization of the dead metal zone, which consequently affects the clarity of our interpretation. To address this, we plan to employ multiple finite element software platforms in subsequent studies to improve visualization capabilities and enable cross-validation of the results.

**Concern #4:** Experimental validation is very limited, with narrow cutting conditions, and results are more illustrative than quantitative; clear visualization of the dead metal zone is missing.

**Author response:** We thank the reviewer for their insightful comments on our manuscript. We fully acknowledge that experimental validation is essential for verifying theoretical predictions. During the initial phase of our study, we systematically compared the slip-line phenomena in simulations and observed that the discrepancy between our theoretical predictions and simulation results became significant when the rake angle exceeded 15°. We therefore concluded that our model is applicable specifically within the 0–15° rake angle range.

It is also worth noting that in practical machining operations, tool rake angles are generally maintained within this range to ensure cutting-edge sharpness and process stability. Given this alignment between theoretical and practical considerations, we focused our experimental validation on this range and did not include larger rake angles.

Regarding the visualization of the dead zone, we acknowledge the technical limitations in capturing high-resolution images of this region under our current experimental conditions. To address this, we plan to employ molecular dynamics simulations in future work to provide clearer insights into the formation and characteristics of the dead zone at the micro-scale.

**Concern #5:** No systematic error analysis or mesh sensitivity study is presented, which reduces the reliability of the simulation findings.

**Author response:** We sincerely thank the reviewer for this professional suggestion. We fully acknowledge the importance of mesh sensitivity analysis for ensuring computational accuracy. In our DEFORM-based cutting simulations, we did observe that different mesh densities affected the results; however, through systematic comparison, the maximum variance was found to be within 5.6%, which falls within an acceptable range for engineering applications. To maintain the focus of the current manuscript, we did not include a detailed mesh sensitivity study here. A comprehensive analysis of this aspect has been provided in the first author's master's thesis, titled "Study on Cutting Force of TC4 Titanium Alloy Based on Slip-Line Field." We will add a citation to this thesis in the revised manuscript for readers interested in the detailed methodology.

[1] Hu Bo. Study on Cutting Force of Titanium Alloy TC4 Based on Slip Line Field [D]. Guizhou University, 2022. DOI:10.27047/d.cnki.ggudu.2022.000441.

**Concern #6:** Literature review is descriptive rather than critical, with insufficient discussion of how the proposed model improves upon or differs from existing approaches.

**Author response:** We thank the reviewer for the valuable suggestion. We have revised the Introduction section accordingly, and the changes have been highlighted in red in the revised manuscript.

**Concern #7:** Language quality and presentation issues reduce clarity; figures are not well-labeled, and overlap exists between background, results, and discussion.

**Author response:** We thank the reviewer for the valuable comments. We have polished the writing throughout the manuscript and systematically checked all figures. Furthermore, we have revised and enhanced specific parts of the Results and Discussion section. All corresponding changes have been highlighted in red in the revised manuscript.

**Concern #8:** Quantitative comparison among theory, simulation, and experiments is weak, leaving the conclusions insufficiently supported.

**Author response:** We sincerely thank the reviewer for this important comment. We fully understand the necessity of strengthening quantitative comparisons to support the conclusions of the paper.

In our previous work, "Mechanical analysis before the steady-state cutting of TC4 titanium alloy," the stress field and cutting force models were systematically validated experimentally. The theoretical derivations in the present study are built directly upon these validated models, and we are confident in the rigor they provide.

We also acknowledge that the current simulation approach is relatively limited, which restricts the depth of our quantitative analysis. In addition, due to constraints in our experimental capabilities, it is challenging to conduct further experimental validation in the short term. To address this, we plan to employ molecular dynamics simulations in future work to investigate the cutting mechanisms of TC4 titanium alloy at the micro-scale, thereby providing cross-scale validation for the theoretical model.

We thank the reviewer again for helping us improve the quality of this manuscript.

**Concern #9**: Overall contribution appears incremental, with insufficient evidence of a breakthrough in understanding slip-line field or cutting mechanisms.

**Author response:** We fully appreciate the importance of groundbreaking advances in a research publication and thank the reviewer for this insightful perspective. While the current study may have limitations in terms of transformative findings, we have developed a follow-up research plan that incorporates more comprehensive theoretical frameworks. Based on this, we have proposed a new direction for studying the slip-line field in TC4 titanium alloy cutting. We believe this direction will offer fresh insights to the field. We thank the reviewer again for helping us improve the scholarly quality of our work.

**Finally,** **thanks again to the editors and reviewers for their valuable comments on our study, which have strongly improved the quality and increased the research contribution of this manuscript. Please do not hesitate to contact us if there are any questions.**
